# Patient and Caregiver Perceptions of Advanced Bladder Cancer Systemic Treatments: Infodemiology Study Based on Social Media Data

**DOI:** 10.2196/45011

**Published:** 2023-03-27

**Authors:** Simon Renner, Paul Loussikian, Pierre Foulquié, Alexia Marrel, Valentin Barbier, Adel Mebarki, Stéphane Schück, Murtuza Bharmal

**Affiliations:** 1 Kap Code Paris France; 2 ICON Lyon France; 3 EMD Serono Rockland, MA United States

**Keywords:** bladder cancer, social media, patient, caregiver, chemotherapy, immunotherapy, qualitative research, cancer treatment, first-line therapy, patient support, adverse event, peer support, cancer, oncology, perception, pharmacotherapy, opinion, attitude

## Abstract

**Background:**

In 2022, it was estimated that more than 80,000 new cases of bladder cancer (BC) were diagnosed in the United States, 12% of which were locally advanced or metastatic BC (advanced BC). These forms of cancer are aggressive and have a poor prognosis, with a 5-year survival rate of 7.7% for metastatic BC. Despite recent therapeutic advances for advanced BC, little is known about patient and caregiver perceptions of different systemic treatments. To further explore this topic, social media can be used to collect the perceptions of patients and caregivers when they discuss their experiences on forums and online communities.

**Objective:**

The aim of this study was to assess patient and caregiver perceptions of chemotherapy and immunotherapy for treating advanced BC from social media–posted data.

**Methods:**

Public posts on social media in the United States between January 2015 and April 2021 from patients with advanced BC and their caregivers were collected. The posts included in this analysis were geolocalized to the United States; collected from publicly available domains and sites, including social media sites such as Twitter and forums such as patient association forums; and were written in English. Posts mentioning any line of chemotherapy or immunotherapy were qualitatively analyzed by two researchers to classify perceptions of treatments (positive, negative, mixed, or without perception).

**Results:**

A total of 80 posts by 69 patients and 142 posts by 127 caregivers mentioning chemotherapy, and 42 posts by 31 patients and 35 posts by 32 caregivers mentioning immunotherapy were included for analysis. These posts were retrieved from 39 public social media sites. Among patients with advanced BC and their caregivers, treatment perceptions of chemotherapy were more negative (36%) than positive (7%). Most of the patients’ posts (71%) mentioned chemotherapy factually without expressing a perception of the treatment. The caregivers’ perceptions of treatment were negative in 44%, mixed in 8%, and positive in 7% of posts. In combined patient and caregiver posts, immunotherapy was perceived positively in 47% of posts and negatively in 22% of posts. Caregivers also posted more negative perceptions (37%) of immunotherapy than patients (9%). Negative perceptions of both chemotherapy and immunotherapy were mainly due to side effects and perceived lack of effectiveness.

**Conclusions:**

Despite chemotherapy being standard first-line therapy for advanced BC, negative perceptions were identified on social media, particularly among caregivers. Addressing these negative perceptions of treatment may improve treatment adoption. Strengthening support for patients receiving chemotherapy and their caregivers to help them manage side effects and understand the role of chemotherapy in the treatment of advanced BC would potentially enable a more positive experience.

## Introduction

In 2022, an estimated 81,180 new cases of bladder cancer (BC) and 17,100 BC-related deaths occurred in the United States [1]. Of these new BC cases, 12% were diagnosed as locally advanced (7%) or metastatic (5%) BC (hereafter collectively referred to as advanced BC). Advanced BC is an aggressive disease with a poor prognosis. In particular, the 5-year survival rate for metastatic BC is 7.7% [1]. BC occurs predominantly in men, accounting for approximately 75% of all cases and deaths [1]. BC is staged according to tumor size, lymph node invasion, and extension of disease. In the early stages, BC is localized within the bladder but may extend beyond the bladder, initially into the adjacent regions and organs; in later stages, BC metastasizes throughout the body [2,3].

Current standard-of-care first-line treatment for advanced BC comprises platinum-based chemotherapy followed by avelumab (immunotherapy) maintenance for nonprogressive disease on chemotherapy [4]. Chemotherapy with cisplatin or carboplatin combined with gemcitabine is recommended [3,4]. Alternatively, other nonpreferred first-line therapies can be used, including atezolizumab in patients not eligible for platinum-based chemotherapy or patients not eligible for cisplatin-containing chemotherapy with tumors expressing programmed death-ligand 1, and more recently, pembrolizumab in patients not eligible for platinum-based chemotherapy [4]. After failure of first-line platinum-based chemotherapy, other therapies such as avelumab, erdafitinib, pembrolizumab, nivolumab, enfortumab vedotin-ejfv, or chemotherapy are approved for use [4].

Patients with BC experience various physical symptoms (including pain, bleeding, and sexual dysfunction, as well as urinary frequency, incontinence, and obstruction) depending on the disease stage [5-7]. BC also provokes significant social, cognitive, functional, and relational problems, as well as emotional distress, including anxiety and depression [5-7]. It is critical that physicians consider the impact of these symptoms on patient quality of life and treatment satisfaction when making therapeutic decisions. Traditionally, symptoms and quality of life data from the patient’s perspective have been collected during clinical trials using standardized patient-reported outcome questionnaires such as the Functional Assessment of Cancer Therapy-General Scale (FACT-G), Functional Assessment of Cancer Therapy-Bladder Symptom Index-18 [8], European Organization for Research and Treatment of Cancer Core Quality of Life 30-item questionnaire (QLQ-C30) [9], and EuroQol 5-level (EQ-5D) [8].

The studies assessing quality of life in cancer have mainly focused on the patient’s perspectives and, to a much lesser extent, on the caregiver’s perspective. This is despite the development of several instruments that were specifically designed to collect data concerning the effect of cancer on the caregiver’s quality of life [10], such as the Caregiver FACT-G [11], the Comprehensive Needs Assessment Tool for Cancer-Caregivers [10,12], and the Quality of Life in Life-Threatening Illness: Family Carer Version [13]. Indeed, few studies have assessed cancer treatment from the caregiver’s perspective [5,10]. In those that did, caregivers reported anxiety, depression, and decreased quality of life. However, information about caregivers may be challenging to collect and analyze in clinical trials due to many factors, including the heterogeneous population, varying levels of involvement in care, and possibility of bias such as caregivers feeling guilty when reporting caregiving as a burden [5].

Another approach to exploring patients’ and caregivers’ perspectives on cancer is to use social media. Social media offer unprompted discussions between patients and caregivers, which may capture more genuine perspectives than traditional surveys, questionnaires, or interviews [14-16]. Social media also allow the collection of data from a much broader, geographically dispersed sample (ie, from a wide range of countries or locations), which may mitigate issues with sample size when examining very specific, nuanced patient groups. Moreover, social media allow patients and caregivers to access communities with other patients, caregivers, and health care professionals. In these communities, patients and caregivers can request information, share experiences, voice concerns, learn about treatments, and connect with others for support [17]. This was particularly evident during the COVID-19 pandemic, which exacerbated the need for online support. Strict social distancing and containment measures isolated patients, and in response, many patients and caregivers began to seek emotional support and information through social media [18]. The provision of an ever-increasing amount of information and communication to these patients and caregivers is a matter of prevention and public health, especially concerning cancer [19,20].

The aim of this retrospective study was to assess patient and caregiver perceptions of advanced BC treatments, specifically any line of chemotherapy or immunotherapy, using data from US social media posts.

## Methods

### Study Design

This retrospective, real-world study retrieved and analyzed data posted by patients and caregivers on social media concerning the treatment of advanced BC. Data posted between January 1, 2015, and March 4, 2021, were considered for the study. Posts on publicly available domains, written in English, and geolocalized in the United States were included. Posts from all public sites, including social media sites such as Twitter and forums such as patient association forums, were included. In contrast, posts on Facebook and Instagram were not included, since not all posts on these sites are publicly available.

### Social Media Content Extraction and Selection

Data (social media posts) were retrieved from publicly available social media sites by identifying and extracting posts, eliminating irrelevant data, and then filtering the posts to obtain only messages concerning advanced BC. The Brandwatch extractor (Cision Ltd, Chicago, IL) software was used to identify all public posts available on the web using combinations of words related to BC (the full query is available in Multimedia Appendix 1). These discussions were extracted with the associated metadata (eg, publication date or country) and anonymized. Irrelevant posts such as those from discussion forums not related to BC, those not pertaining to patients or caregivers, and those not featuring advanced BC were then eliminated by applying a three-step process.

Initially, posts from irrelevant sources such as potential advertising sites or forums related to pets and animals were removed. Then, a machine learning algorithm was applied to the data set. The algorithm recognized three different variables (lexical field, syntactic aspects of the post, and semantic style) to identify and classify patients and caregivers according to their respective vocabulary and grammar. Next, a manual review was performed to remove inconsequential posts unrelated to patient and caregiver perceptions. Finally, the messages were filtered using keywords characteristic of advanced BC (eg, stage IV BC or terminal BC). Once these relevant posts had been identified, the users or usernames associated with these posts were considered to be directly concerned with advanced BC. Thus, all messages from these users in the data set mentioning BC were retained, even if they did not mention advanced BC.

The algorithm used in this study was previously developed using a training set of 12,330 messages related to different health domains (eg, dermatology, tobacco use, and oncology). The method consists of a pipeline featuring two extreme gradient boosting [21] classifiers (one for caregivers’ experiences and one for patients’ experiences) applied successively. This method allowed identification of whether a post belonged to a patient, a caregiver, or neither. Both classifiers were based on features combining pronouns and lexical fields describing relatives and pathologies (eg, “my [pronoun] father [relative] has cancer [pathology]”). We trained the algorithm by first identifying the caregivers; this was carried out on the whole data set. To determine patients’ messages, we then reapplied the algorithm on the rest of the data set (excluding the already identified caregiver messages). Evaluation of performances yielded F1-scores (a measure of accuracy combining precision and recall) of 88.0% and 87.0% for the caregiver and patient classifier, respectively. In this work, manual review following the application of the algorithm ensured validation of the results. Prediction mistakes were corrected by the annotator.

The data sets corresponding to the patients and caregivers were then filtered using keywords associated with cancer therapy, such as “chemotherapy” and “immunotherapy.” The complete list of search terms is available in Multimedia Appendix 1. Posts containing both “chemotherapy” and “immunotherapy” were classified in both therapeutic categories.

### Data Analysis

#### Age and Sex

When possible, the age and sex of the patient/caregiver were determined by a manual review of the messages (eg, “My 56 [year old] husband has stage 4 bladder cancer”). Otherwise, the data for age and sex were coded as “undetermined.”

#### Qualitative Analysis

Qualitative analysis was based on the manual annotation of caregiver or patient posts by two independent analysts (PL and SR). Annotation guidelines were agreed on prior to analysis. This manual analysis aimed to identify the BC treatments used, treatment modalities, patient or caregiver perceptions of treatments, and disadvantages or benefits of the treatments.

#### Treatment Characteristics

The manual analysis identified data characterizing the systemic treatment of advanced BC. The posts were used to determine the treatment and whether the treatment was administered or taken, based on the messages (eg, “[…] I never heard of [treatment]. I will have to look into that” or “[…] he did 7 rounds of chemo”). Data concerning the chemotherapy and immunotherapy administered, including numbers of cycles and duration of treatments, were collected.

#### Treatment Perceptions

Treatment perception was evaluated through manual analysis. Depending on the message posted by patients or caregivers, the treatment perception was classified as positive, negative, mixed, or no perception. A positive opinion of a treatment, such as posts mentioning that the treatment was effective or that the BC had stabilized, were classified as having a positive perception (eg, “Highly recommend [treatment]”). A poor treatment perception, such as indicating that treatment was unsuccessful or had significant side effects or that the disease relapsed, was classified as a negative perception (eg, “[…] chemo didn’t work”). Mentions of both positive and negative expressions were also analyzed and classified as mixed. Messages without treatment perception (eg, “I’ve been on [treatment] since April”) were classified as no perception. The disadvantages and benefits of treatments associated with treatment perception were also collected.

### Ethical Considerations

This study used data from publicly available sources; thus, private groups or web pages were not included in our data extraction process. We did not seek permission since users automatically consent to the reuse of their data when they post on public platforms. Moreover, the study’s findings contain no identifiable information and are presented in aggregate. Names, usernames or handles, geographic locations, and any other sensitive data were not included.

## Results

### Identification of Posts With Treatment Mentions

**Figure 1:**
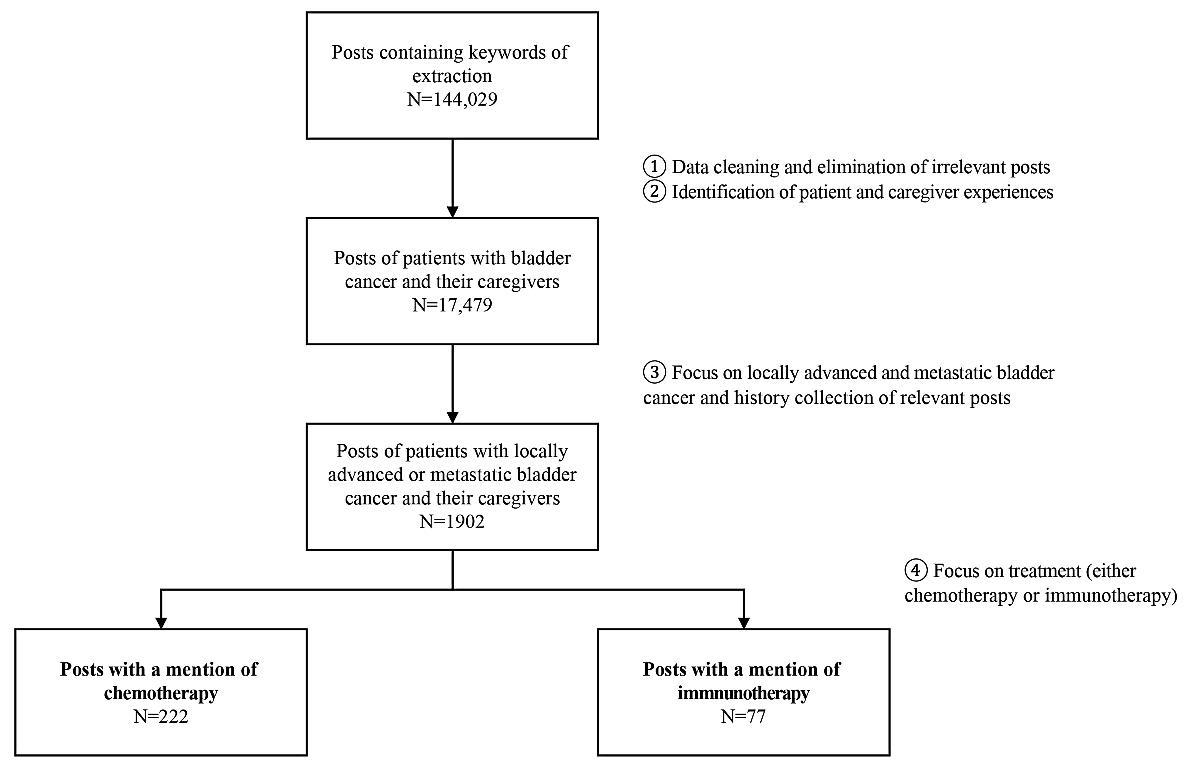
Post identification and selection.

Advanced BC treatments, either chemotherapy or immunotherapy, were mentioned in 299 posts; 222 mentioned chemotherapy and 77 mentioned immunotherapy (Figure 1).

### Population

The details of the posts mentioning treatments by patients and caregivers are described in Table 1. There were 80 posts by 69 patients and 142 posts by 127 caregivers mentioning chemotherapy from 38 discussion sources (Table 1). In addition, there were 42 posts by 31 patients and 35 posts by 32 caregivers mentioning immunotherapy from 13 discussion sources (Table 1). The forums and social media sites where patients and caregivers discussed specific BC treatments are described in Table 2.

Among the 222 posts mentioning chemotherapy, only 21 of 69 patients (30%) and 35 of 127 caregivers (28%) mentioned an age. In addition, 40 patients (58%) and 121 caregivers (95%) indicated a sex. Of the 40 patients with known sex, 20 (50%) were female and 20 (50%) were male, whereas of the 121 caregivers with known sex, 87 (72%) were male and 34 (28%) were female. Among the 77 posts mentioning immunotherapy, 18 of 31 patients (58%) and 30 of 32 caregivers (94%) indicated a sex, while 11 of 31 patients (35%) and 8 of 32 caregivers (25%) mentioned an age (Table 1). For internet users with a known sex, the majority were male for both patients (12/18, 67%) and caregivers (21/30, 70%).

**Table 1:** Characteristics of patients and caregivers who posted social media messages.

Characteristics			Patients					Caregivers				
			Chemotherapy		Immunotherapy			Chemotherapy			Immunotherapy	
Users, n			69		31			127			32	
Posts, n			80		42			142			35	
**Social media users, n (%)**												
	Bladdercancersupport.org	23 (33.3)		4 (12.9)			24 (18.9)			8 (25.0)		
	Twitter	9 (13.1)		5 (16.1)			20 (15.8)			3 (9.4)		
	Inspire.com	21 (30.4)		17 (54.8)			8 (6.3)			3 (9.4)		
	Reddit	6 (8.7)		2 (6.5)			21 (16.5)			8 (25.0)		
	Others	10 (14.5)^a^		3 (9.7)^b^			54 (42.5)^c^			10 (31.2)^d^		
**Sex, n (%)**												
	Female	20 (29.0)		6 (19.4)			34 (26.8)			9 (28.1)		
	Male	20 (29.0)		12 (38.7)			87 (68.5)			21 (65.6)		
	Undetermined	29 (42.0)		13 (41.9)			6 (4.7)			2 (6.3)		
**Age (years), n (%)**												
	<40	4 (5.8)		2 (6.5)			2 (1.6)			0 (0)		
	40-59	7 (10.1)		2 (6.5)			12 (9.4)			1 (3.1)		
	≥60	10 (14.5)		7 (22.5)			21 (16.6)			7 (21.9)		
	Undetermined	48 (69.6)		20 (64.5)			92 (72.4)			24 (75.0)		

^a^These 10 patients expressed themselves on eight other forums such as cancer.org, navigatingcancer.com, or ic-network.com.

^b^These three patients expressed themselves on three other forums (cancer.org, cafemom.com, and delphiforums.com).

^c^These 54 caregivers expressed themselves on 28 other forums such as cancer.org, cancercompass.com, or babycenter.com.

^d^These 10 caregivers expressed themselves on seven other forums such as cancer.org, healingwell.com, or cancercompass.com.

**Table 2:** Forums and social media where users mentioned specific bladder cancer treatments.

Forum	Chemotherapy posts, n	Immunotherapy posts, n
Bladdercancersupport.org	49	13
Twitter	35	10
Inspire.com	32	24
Reddit	30	12
Cancer.org	15	6
Cancercompass.com	6	0
Navigatingcancer.com	5	0
Other forums	50^a^	12^b^

^a^Sources with fewer than 5 posts, 31 additional forums.

^b^Sources with fewer than 5 posts, 8 additional forums.

### Treatments

#### Chemotherapy in Any Line of Treatment

Overall, 222 posts mentioned chemotherapy; 80 (36%) of these were posted by patients and 142 (64%) were posted by caregivers. Analysis of patient posts revealed that 87% of patients had undergone chemotherapy. Furthermore, 74 patient and caregiver posts mentioned chemotherapy administration. The numbers of chemotherapy cycles taken or planned were expressed in 39 posts by patients or caregivers. The numbers of chemotherapy cycles most frequently reported were four cycles in 12 posts (31%), three cycles in eight posts (21%), and six cycles in six posts (15%). The duration and frequency of chemotherapy were discussed in 10 of 222 posts (5%) by patients or caregivers. Most patients had chemotherapy once a week.

**Figure 2:**
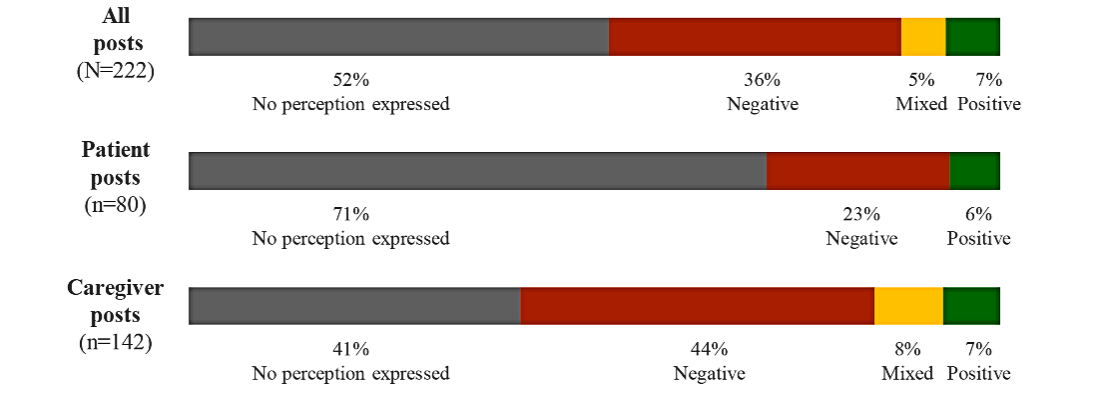
Overall perception of chemotherapy.

Table 3 provides some examples of posts describing patient and caregiver perceptions of chemotherapy. Concerning chemotherapy, 71% of patient posts and 41% of caregiver posts expressed no perception. Among the caregiver posts, 44% were negative, 8% were mixed, and 7% were positive. Overall, among both patients and caregivers, 36% of posts were negative and 7% were positive (Figure 2). Among patient and caregiver posts containing positive comments about chemotherapy, 19 mentioned the perceived benefits, of which 13 (68%) were related to the effectiveness of chemotherapy. Patients and caregivers expressed effectiveness generally, without going into detail; two posts expressed the opinion that chemotherapy allowed patients to live longer.

The disadvantages of chemotherapy were mentioned in 87 of 222 posts (39%). Patients with BC and their caregivers were most commonly burdened by side effects in 30 of 87 posts (34%). Chemotherapy being ineffective was mentioned in 29 of 87 posts (33%). Indeed, after initial promising results during the first cycles of chemotherapy, patients and caregivers reported a decline in effectiveness or ineffectiveness with further cycles, leading to a change in treatment when possible. Not being eligible to start or continue chemotherapy was considered a disadvantage for which patients and caregivers expressed disappointment or frustration in 12 of 87 posts (14%). Indeed, some patients could not start or continue chemotherapy because it was contraindicated, they did not meet the treatment criteria (mainly in clinical trials), and/or they were not considered fit enough for chemotherapy.

**Table 3:** Examples of posts by patients and caregivers about chemotherapy.^a^

Characteristics	Example of post
Number of chemotherapy cycles	I have stage 4 bladder cancer. I was given 6 months. Did *7 rounds of chemo*^b^ […] [Patient]
Duration and frequency of chemotherapy	He’s scheduled to have *chemo once a week*, let’s see what happens. [Caregiver]
No perception expressed	[…] My wife has Stage 4 Bladder Cancer. She is going through what the Oncologist refer to as an ‘Aggressive’ schedule of Chemo. Two days back to back of MVAC^c^. She had her first two days this Tuesday and Wednesday. [Caregiver]
Negative perception because of side effects	[…] *The weeks that I’m on cisplatin [are] the worst, mostly fatigue and upset stomach. […] My worst side effects occur on days 2-4 of my treatment, so I’m over it and ready to gorge myself on day 5.* […]. [Patient]
Positive perception with a good response	Glad to know the great team & really *glad to be a stage 4 bladder cancer patient that responded to chemo.* [Patient]

^a^This table describes some representative patient/caregiver perceptions verbatim that were observed on social media, but any conclusions on safety or efficacy of treatments cannot be inferred from them.

^b^Italicized text indicates specific text relevant to the characteristic.

^c^MVAC: methotrexate, vinblastine sulfate, doxorubicin hydrochloride (Adriamycin), and cisplatin.

#### Immunotherapy in Any Line of Treatment

Overall, 77 posts mentioned immunotherapy, 42 (55%) of which were from caregivers. Of the 35 patients who posted messages, 31 (89%) had received immunotherapy. Details regarding immunotherapy administration were mentioned in 18 of 77 posts (23%). The numbers of administered or planned rounds (ranging from three to eight) were stated in 4 of 77 posts (5%). Immunotherapy duration and frequency were mentioned in 6 of 77 posts (8%). The immunotherapy administration modalities appeared in 18 of 77 posts (23%). Most immunotherapies mentioned in the posts lasted for more than 1 year and were most often administered once every 3 weeks. Table 4 provides some examples of posts describing patient and caregiver perceptions of immunotherapy.

**Figure 3:**
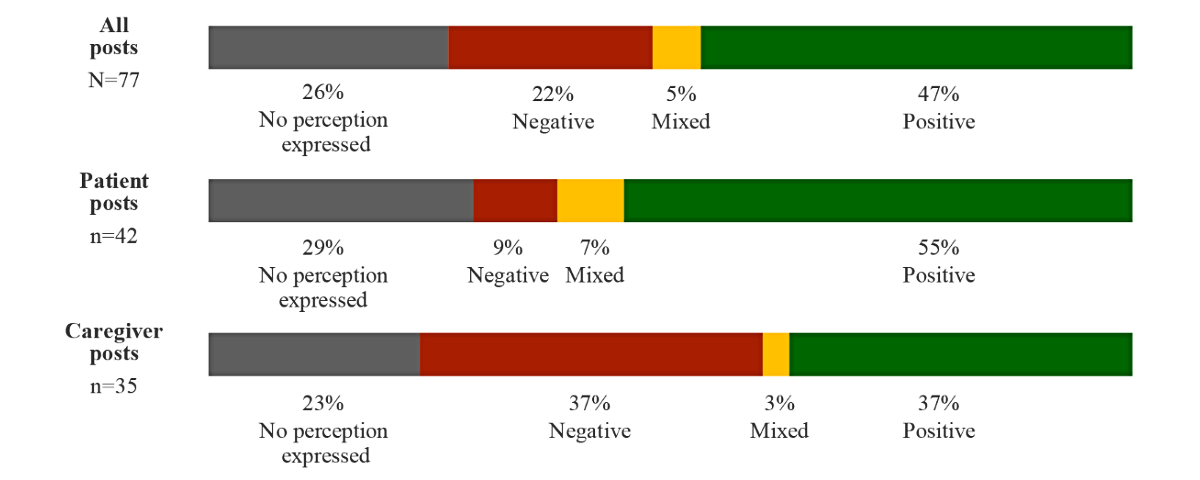
Overall perception of immunotherapy.

Immunotherapy was perceived positively in 36 of 77 posts (47%), while 17 of 77 (22%) posts perceived immunotherapy negatively (Figure 3). The perception of immunotherapy was negative in 13 of 35 (37%) caregiver posts and in 4 of 42 (10%) patient posts. Benefits of immunotherapy were cited in 25 posts (patients or caregivers), including treatment efficacy in 10 (40%), few side effects in 8 (32%), and prolonged life in 2 (8%) posts.

The disadvantages of immunotherapy were mentioned in 25 posts by patients or caregivers. The major disadvantages were perceived lack of effectiveness in 12 of 25 posts (48%) and presence of side effects in 10 of 25 posts (40%). Patients or caregivers described persistent sequelae after immunotherapy in 2 of 25 posts (8%).

**Table 4:** Examples of posts by patients and caregivers about immunotherapy.^a^

Characteristics	Example of post
Data about administration	I have been on immunotherapy for this for about *16 months now*^b^ and am expecting my 2nd child any day now! [Patient] Interestingly this is the immunotherapy they are giving my elderly uncle with metastatic bladder cancer, *it’s every 3 weeks one week off, in another week and half he gets his 2nd treatment.* [Caregiver]
Positive perception	If so I just want you to know that [immunotherapy] *an immunotherapy drug caused my metastatic lymph nodes to disappear in 2 weeks. […] the life saving [immunotherapy] is keeping the cancer that would kill me sooner at bay.* [Patient] […] *I was given [immunotherapy]. I am now in remission!!!!! There is hope!* Immunotherapy can be given should anything return and *so far side effects are minimal!!!* FINALLY!! [Patient]
Negative perception because of side effects or perceived lack of effectiveness	[…] I’ve been on [immunotherapy] since April after chemo didn’t work. *It wasn’t too bad at the beginning, itching, dizziness and fatigue, but the latest couple of treatments have left me with sore aching muscles and joints which is one of the less common side effects.* [Patient] My husband […] is currently taking immunotherapy […] *which has had numerous side effects like loss of taste buds and loss of the adrenal and pituitary glands.* [Caregiver]
Negative perception because of persistent sequelae	I was able to travel to [cancer center] and join a clinical trial, and then another trial and finally a third trial of [immunotherapy] and [immunotherapy] which seems to be working for the cancer *but which destroyed my lungs.* [Patient]

^a^This table describes some representative patient/caregiver perceptions verbatim that were observed on social media, but any conclusions on safety or efficacy of treatments cannot be inferred from them.

^b^Italicized text indicates specific text relevant to the characteristic.

## Discussion

### Principal Findings

To our knowledge, this is the first study to examine perceptions of advanced BC systemic treatments in social media posts by patients and their caregivers. Despite recent therapeutic advances for advanced BC, little is known about patient and caregiver perceptions of these therapies. Our results provide valuable insights into their perceptions. Concerning chemotherapy, we found that 71% (n=57) of patient posts expressed no perceptions. They described chemotherapy objectively, as an inevitable part of their health care journey. In contrast, caregivers were more likely to express their opinion of chemotherapy, with 44% (n=62) of their posts being negative, 8% (n=11) mixed, and 7% (n=10) positive. Only 9% (n=19) of all posts contained positive perceptions about chemotherapy, and these were mainly focused on effectiveness. Chemotherapy disadvantages were discussed in 39% (n=87) of posts and were mostly related to side effects and perceived lack of effectiveness. Conversely, patients expressed their opinions about immunotherapy, with 55% (n=23) of posts being positive and 29% (n=12) expressing no perceptions. Positive comments focused on treatment effectiveness, few side effects, and extending the patient’s life. Interestingly, caregivers were more likely to express a negative perception about immunotherapy than patients, accounting for 37% (n=13) and 10% (n=4) of the posts, respectively. Negative perceptions about immunotherapy focused on perceived lack of effectiveness, side effects, and persistent sequelae.

In our study, more patients and caregivers shared their perceptions of chemotherapy (222 posts) than immunotherapy (77 posts). This is expected since platinum-based chemotherapy has been the preferred standard first-line treatment for patients with advanced BC for a long time [3,4]. Furthermore, immunotherapy was only authorized as part of the advanced BC treatment pathway in 2016; therefore, during the first 2 years of the study, chemotherapy was the only treatment option for advanced BC. More positive perceptions were noted for immunotherapy among overall posts (patients and caregivers), possibly because these are newer treatments with favorable safety profiles and their increased use in advanced BC has received positive press, including the recent positive results reported with avelumab as the new standard of care in first-line maintenance of advanced BC [22].

Among caregiver posts, a mostly negative perception of advanced BC treatments was revealed. This negative perception may be explained by the fact that caregivers often feel poorly equipped to support patients, with limited knowledge about BC and treatments [6,23]. Furthermore, treatment side effects severely impact both patient and caregiver quality of life and can be expected to negatively influence treatment perceptions [24]. Considering the pivotal role that many caregivers assume in the lives of patients with BC and the importance of their involvement in patient care, their level of understanding should be acknowledged by clinicians and other members of the multidisciplinary care team. It is thus crucial that caregivers be informed and provided with the support required to effectively assist patients with their cancer treatments.

While patients with BC may use social media to share their experiences, there is a paucity of literature using social media data to gauge patient perceptions [25]. Overall, we found that caregivers engaged more frequently and actively on social media than patients. These results are consistent with a recent systematic review in which the authors noted that most patients with BC were older men with lower electronic literacy [25]. Therefore, it is the caregivers, on behalf of patients, who may be actively engaging on social media to obtain further information. The increased social media presence of caregivers could also be due to the severe grief or burden related to end-of-life care that they experience, with messages often posted several years after the patient’s death [26]. Interestingly, most caregivers identified in the Renner et al [26] study were women, who have been found to seek emotional support in online health communities more often than men [26,27].

### Study Strengths and Limitations

This study design has several strengths. A large sample size collected over a 6-year period was analyzed. The results include data from a variety of social media sources and could provide another dimension to research on treatment perceptions. Accessing publicly available social media data is quick, inexpensive, and has no access restrictions.

However, our innovative research approach does entail several limitations. The posts extracted were limited to publicly available sites, which excluded popular social media networks such as Facebook and Instagram, meaning that many data were not included. Furthermore, relevant posts may have been inadvertently discarded during the filtering process. Duplication may have also been possible if users were active on more than one forum. Additionally, our analysis is based on the spontaneous declarations of internet users about their experience of the disease or their treatment. Although this type of data collection allows us to be representative of the population of internet users that post on social media, it is not necessarily representative of the general population.

A further limitation with using social media posts is that posts only have limited information. Critical information to place the post in context (such as the disease stage or treatment details) may be missing. This lack of data also makes it difficult to compare our results with those of traditional epidemiological studies. Furthermore, few forum users shared demographics such as age, sex, and location in the publicly accessible data that were used for this study, making it impossible to judge whether the data are representative of patients with advanced BC and their caregivers in the United States. The data quality depends on patient and caregiver electronic literacy, their experiences and perceptions, and their capacity to understand and accurately communicate BC information, including the type or stage of BC and treatment administered. Patients and caregivers do not necessarily include all details about their treatment, such as type of treatment, duration, lines of treatment, and response information. These self-reported data may be subject to recall bias. In addition, we cannot verify the authenticity of the published posts.

It is also possible that since the data came from social media, posts may be more negative [28,29]. Twitter has more anonymity than sites such as Facebook, meaning that more negative behavior could be provoked [30,31]. Since most of our data came from Twitter, this could partly explain our findings. Finally, our study is prone to selection bias, as included patient and caregiver posts may not represent all patients with BC and their caregivers. Indeed, engagement with social media depends on age and sex, ethnicity, socio-professional class, and income, as well as levels of education and technological and health literacy.

### Future Work and Impact on Care

This study revealed areas that need to be addressed. Patients and caregivers indicated that they lacked information about patient experiences with advanced BC and its treatments. This is consistent with the fact that studies on social media reported that BC remains underrepresented online compared with other cancers [25,32]. There is therefore a need for clear, accurate, and accessible information about BC treatments for patients and caregivers.

Currently, chemotherapy is the recommended first-line treatment for patients with advanced BC. The negative perception of chemotherapy identified in this study needs to be investigated and considered, as it may influence the choice of treatment of patients seeking advice in social media forums. Therefore, a reflection work could be initiated in partnership with physicians who treat patients with advanced BC. This reflection work could help to identify the levers of improvement and communication to best manage the potential stress and anxiety associated with chemotherapy for patients and caregivers. Subsequently, it would be interesting to study the impact of chemotherapy perception on the adherence to treatment and the quality of life of patients and caregivers using social media [33].

This study also highlights that social media posts from patients and caregivers may provide real-world insights into treatment perceptions and quality of life, as previously shown in other studies [34]. It would also be interesting to cross-reference this or a future study applying our methodology with other qualitative studies on patients with advanced BC to compare the different signals and analyze their potential complementarity [16]. The extension of our research method to other countries or regions may be also valuable to identify initiatives that could improve treatment perceptions, quality of care, and quality of life for patients with BC and their caregivers.

### Conclusion

Real-life data from social media posts may generate further insights into the impact of BC treatments on patients and caregivers not captured in standardized clinical study questionnaires. In advanced BC, chemotherapy remains the cornerstone of first-line therapy. Despite this, there appear to be some negative perceptions of chemotherapy among patients with advanced BC and more so among their caregivers. Addressing these negative perceptions of treatment may improve treatment adoption. Additional support and information could be offered to patients and their caregivers on BC therapy and how to manage side effects. This may allow them to have a more positive experience, which has increased importance given the survival benefits associated with first-line platinum-based chemotherapy followed by avelumab maintenance in those whose disease has not progressed on chemotherapy.

## Appendix

Multimedia Appendix 1 Query used for extraction on Brandwatch and keywords used for the focus on treatment.

## Abbreviations

BC: bladder cancer
EQ-5D: EuroQol 5-level
FACT-G: Functional Assessment of Cancer Therapy-General Scale
QLQ-C30: European Organization for Research and Treatment of Cancer Core Quality of Life 30-item questionnaire

## Data Availability

The derived data sets generated and analyzed during this study are not publicly available due to Kap Code’s private ownership of this database. Indeed, these public social media data have been extracted, cleaned, and consolidated in a database using Kap Code tools and are not found anywhere else in this format. If, however, this database is to be reused in a future study, a partnership with Kap Code could potentially take place. For this, please contact the Kap Code team at the following email address: hello@kapcode.fr
