# Supplementary material for: Patient and Caregiver Perceptions of Advanced Bladder Cancer Systemic Treatments: Infodemiology Study Based on Social Media Data
Source: JMIR Cancer. 2023 Mar 27;9:e45011. doi: 10.2196/45011 (PMC10131927; doi:10.2196/45011)
Supplement: Multimedia Appendix 1 [file cancer_v9i1e45011_app1.docx]

**Multimedia Appendix 1**

**Query used for extraction on Brandwatch:**

((cancer OR carcinoma OR tumor) NEAR/3 (bladder OR urothelial OR urotelial)) OR bavencio OR avelumab OR (bc NEAR/6 (bladder OR uro*))

**Keywords used for our focus on treatment:**

| Keywords | Data set |
| --- | --- |
| Atezo | Immunotherapy |
| Atezolizumab | Immunotherapy |
| Avelumab | Immunotherapy |
| Bavencio | Immunotherapy |
| Enfortumab | Immunotherapy |
| Enfortumab vedotin | Immunotherapy |
| EV | Immunotherapy |
| Immunetherapy | Immunotherapy |
| Immune-therapy | Immunotherapy |
| Immuno | Immunotherapy |
| Immuno oncology | Immunotherapy |
| Immuno-oncology | Immunotherapy |
| Immunotherapy | Immunotherapy |
| imunetherapy | Immunotherapy |
| Imune-therapy | Immunotherapy |
| imuno | Immunotherapy |
| Imuno oncology | Immunotherapy |
| Imuno-oncology | Immunotherapy |
| imunotherapy | Immunotherapy |
| IO | Immunotherapy |
| Ketruda | Immunotherapy |
| Keytruda | Immunotherapy |
| Lirilumab | Immunotherapy |
| Lirrilumab | Immunotherapy |
| Maintenance | Immunotherapy |
| Nivo | Immunotherapy |
| Nivolumab | Immunotherapy |
| Opdivo | Immunotherapy |
| Pembro | Immunotherapy |
| Pembrolizumab | Immunotherapy |
| Penbro | Immunotherapy |
| Penbrolizumab | Immunotherapy |
| Tecentriq | Immunotherapy |
| Carbo | Chemotherapy |
| Carbo platin | Chemotherapy |
| Carboplatin | Chemotherapy |
| Chemo | Chemotherapy |
| Chemotherapy | Chemotherapy |
| Cisplatin | Chemotherapy |
| Etoposide | Chemotherapy |
| Gem | Chemotherapy |
| Gemcitabin | Chemotherapy |
| Gemzar | Chemotherapy |
| MVAC | Chemotherapy |
| Platin | Chemotherapy |
| Platine | Chemotherapy |
| Platinum | Chemotherapy |
